# Living with PCOS: A Narrative of its Biology, Diagnosis, and Evolving Treatment

**DOI:** 10.2174/0118715303390700251003050039

**Published:** 2025-10-27

**Authors:** Samya Shams, Shagufta Jawaid, Nisha Rai, Kanishk Kala, Anjali Choudhary, Nauroz Neelofar, Firoz Anwar

**Affiliations:** 1 Department of Pharmacy Practice, School of Pharmaceutical Sciences, Shri Guru Ram Rai University, Dehradun, Uttarakhand, India;; 2 Department of Obstetrics and Gynae, Shri Guru Ram Rai Institute of Medical and Health Sciences, Patel Nagar, Dehradun, Uttarakhand, India;; 3 Department of Obstetrics and Gynae, Himalayan Institute of Medical Sciences, Swami Rama Himalayan University, Jollygrand, Dehradun, Uttarakhand, India;; 4 Department of Biochemistry, Faculty of Sciences, King Abdulaziz University, Jeddah, Saudi Arabia

**Keywords:** Polycystic ovary syndrome, endocrine disorder (Hyperandrogenism), insulin resistance, metabolic syndrome, emerging therapies, AI-driven evaluation

## Abstract

**Introduction:**

Polycystic ovary syndrome (PCOS) is a complex endocrine disorder characterized by hyperandrogenism, ovarian dysfunction, and metabolic abnormalities. It affects between 4% and 21% of females of fertile age. The present review provides a comprehensive analysis of PCOS, covering pathophysiology, diagnostic criteria, prevalence, biochemical markers, and demographic influences. It examines genetic, hormonal, and lifestyle variables, contributing to the conditions, highlighting ethnic disparities in prevalence.

**Methods:**

To systematically analyse current data and refine evidence-based diagnosis and management guidelines, published in 2018, a global team was assembled. A systematic literature search was conducted across major academic databases, including PubMed, Scopus, Web of Science, and Google Scholar, to identify relevant studies on PCOS pathophysiology, diagnosis, and emerging therapies. Based on the global team's view, our generalised narrative review explores metabolic implications such as glucose intolerance, obesity, and type-2 diabetes, as well as hormonal imbalances involving androgens, luteinizing hormone, and prolactin.

**Results:**

Lifestyle factors, socioeconomic status, and ethnicity significantly influenced PCOS expression. Advancements in diagnosis, including AI-driven evaluation and precision medicine approaches, are discussed, alongside improvements in biochemical testing and treatment strategies.

**Discussion:**

PCOS expression varies by lifestyle, ethnicity, and socioeconomic status. Emerging diagnostics like AI and precision medicine enhance detection, treatment, and understanding of its metabolic complexity.

**Conclusion:**

The review emphasizes the importance of personalized interventions and integrated healthcare approaches to enhance PCOS management and patient outcomes.

## 1 INTRODUCTION

The hyperandrogenic state identified as polycystic ovary syndrome (PCOS) is linked to polycystic ovarian morphology and persistent oligo-anovulation [1]. In addition to metabolic abnormalities, including an excess amount of insulin, which are known as key contributors to altered androgen production and metabolism, it is frequently linked to psychological impairments, such as depression and other mood disorders [2]. The majority of PCOS-affected women have more weight as compared to unaffected individuals [3], resulting in a high level of androgen release, although cooperating metabolic and reproductive processes and possibly contributing to the phenotypic progression of PCOS [4]. Adrenal and ovarian androgen secretion increases, polycystic ovaries, irregular menstruation, and hyperandrogenic metabolic syndrome symptoms, such as alopecia, acne, and/or hirsutism, are the hallmarks of PCOS [5]. PCOS is a metabolic disease in addition to a reproductive endocrinopathy [6]. Insulin resistance, which encompasses the disorders of hyperinsulinemia and decreased glucose sensitivity, is one of the most noticeable metabolic signs of PCOS [7]. Hence, the present study is further necessary due to varying existing diagnostic criteria that vary significantly across clinical guidelines, leading to inconsistencies in identification and treatment. Moreover, PCOS is not only a reproductive disorder but also a metabolic and psychological condition, with long-term health implications including type 2 diabetes, cardiovascular disease, and mental health disorders.

## 2 METHODOLOGY

**Fig. (1):**
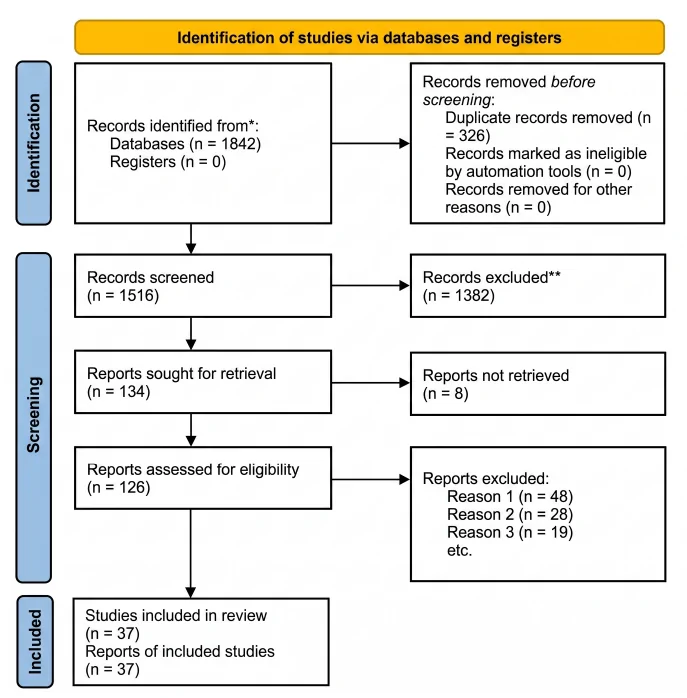
PRISMA flow diagram.

A comprehensive literature search was performed using PubMed, Scopus, Web of Science, and Google Scholar, with a specific emphasis on identifying studies related to the pathophysiology, diagnosis, and treatment of PCOS. The search used Medical Subject Headings (MeSH) terms and keywords such as “Polycystic Ovary Syndrome”, “Endocrine disorder (Hyperandrogenism), (Insulin Resistance)”, “Women”, “Metabolic Syndrome”, and “Emerging Therapies”. Boolean operators refined results, and filters restricted studies to peer-reviewed, English language, human health subject research published between 2014 and 2025. Inclusion criteria encompassed original research, systematic reviews, and meta-analyses focusing on PCOS diagnosis, biochemical or genetic factors, metabolic implications, and treatments, while exclusion criteria removed pre-2014 studies, animal research, case reports, and retracted articles. The study's selection involved title/abstract screening, full text review, and data extraction using standardized forms. Quality assessment was made by a one of third reviewer among us, extracted data included study characteristics, key findings, and methodological rigor. No meta-analysis was performed to summarize the narrative output of the search. The review adhered to PRISMA guidelines, ensuring transparency and reproducibility without direct human or animal involvement (Fig. **1**).

## 3 PREVALENCE AND GLOBAL DEMOGRAPHIC DISTRIBUTION

PCOS affects approximately 4% to 21% of women of reproductive age worldwide, making it one of the most common endocrine disorders in females and a leading cause of infertility [8]. Depending on demographics and diagnostic criteria, PCOS prevalence in India ranges from 2 to 35% [9]. The phenotype of PCOS varies with age [10]. Young PCOS women are particularly concerned about clinical and biochemical hyperandrogenism, and as they age, their metabolic burden tends to rise [11]. While BMI demonstrates its critical involvement in the genesis of the majority of the metabolic sequelae in PCOS, regardless of age, some cardiovascular risk variables are reliant on FAI and SHBG levels [12]. In a cross-sectional research of 9824 females aged 18 to 40, the biased national prevalence of PCOS was 7.2% and 19.6%, respectively, based on the NIH's 1990 and Rotterdam 2003 definitions [13]. PCOS prevalence and incidence according to age worldwide remained 30.7 and 867.7 per 100,000, respectively, according to data from 2021. These figures represent rises to 26.77 percent plus 28.21 percent since 1990. In addition, the standardized age impaired-altered lifecycle years decreased by 27.58% from 1990 to 7.6 per 100,000 worldwide in 2021. Depending on the country, the age-standardized prevalence of PCOS ranged from 93.1 to 3978.9 instances per 100,000 women; Italy had the highest rates (3978.9), followed by Japan (3104.7), as well as New Zealand (2789.7). It is interesting to note that ladies between the ages of 15 and 19 have the highest frequency of PCOS globally. Areas having great standardized incidence (SDI) had the greatest rates of PCOS prevalence (1720.7), SDI with age (70.2), and mortality (15.2) [14]. In Asia, particularly India, a few studies have been carried out to ascertain the prevalence of PCOS and related symptoms [15]. Using the Rotterdam criteria, the study discovered that 4.21% of people had PCOS. The findings also showed that women who lived in cities were more likely than those who lived in rural areas to have PCOS [16]. For the reason of sedentary lifestyles as well as ingestion behaviors, urban dwellers might be more likely to have PCOS, which could ultimately increase their risk for the prolonged illness [17]. Analogously, Bharati and colleagues previously conducted a cross-sectional analysis of a population selected at random in Chennai, Tamil Nadu, India, to determine and compare the prevalence of PCOS in females living in rural and urban areas. The occurrence rate 6% was established using the Rotterdam criteria. Additionally, the study discovered that the risk of PCOS is 0.1 times higher for urban women than for rural ones. Furthermore, it has been demonstrated that stress is a major factor in both initiating symptoms and promoting the growth of PCOS [18].

## 4 PATHOPHYSIOLOGY AND BIOCHEMICAL FACTORS AFFECTING PCOS

PCOS results from hormonal imbalances, including hyperandrogenism and insulin resistance, disturbing the ovarian functions [19]. Increased luteinizing hormone (LH) and reduced follicle-stimulating hormone (FSH) contribute to anovulation and cyst formation [20]. Biochemical factors, including elevated levels of insulin, promote excess androgen production by ovarian theca cells [21], worsening symptoms like hirsutism and menstrual irregularities. Insulin resistance and hyperandrogenism reinforce each other, worsening PCOS manifestations and metabolic complications, creating a cycle of hormonal and biochemical dysregulation. Fig. (**2**) details the complex interplay contributing to PCOS, involving hormonal instability, insulin resistance, free androgens & Insulin-like Growth Factor 1(IGF-1), polycystic ovaries, and hypothalamic-pituitary-ovarian axis dysfunction, all leading to anovulation, infertility, irregular menstruation, acne, and hirsutism.

### 4.1 Altered Biochemical (Hormonal) Parameters in PCOS

Hormonal imbalance in females disrupts essential endocrine functions, affecting menstrual cycles, fertility, and metabolism [22]. Conditions along with PCOS, thyroid dysfunction, and insulin resistance contribute to metabolic disorders [23, 24], leading to weight fluctuation, irregular periods, infertility, and increased risk of diabetes and CVD [25]. Managing hormonal levels along with lifestyle and diet can help in the restoration of hormonal balance.

Testosterone, a major contributing hormone studied *via* CYP11A, the gene encoding P450scc, a crucial enzyme in the synthesis of steroids, is one of the most compelling biological possibilities for susceptibility to polycystic ovarian syndrome (PCOS) [26]. Four of five published studies that searched for evidence of linkage and/or correlation with CYP11A found significant relationships with serum testosterone levels and/or polycystic ovarian (PCO) status [27]. Research investigation showed the DHEAS/free testosterone (DHEAS/FT) ratio (300/5) affected metabolic parameters in women with and without PCOS [28]. Findings demonstrated that females with PCOS who have increased DHEAS/FT ratios have better metabolic phenotypes, but those with low ratios are more likely to experience metabolic problems [29].

The luteinizing hormone to follicle-stimulating hormone (LH/FSH) ratio exhibits a strong association with anti-Müllerian hormone (AMH), a biomarker reflective of ovarian follicular reserve, thereby emphasizing its neuroendocrine relevance in the pathophysiology of lean polycystic ovary syndrome (PCOS) [30]. Mechanistic pathway analyses have demonstrated that AMH modulates the LH/FSH ratio by serving as a mediator in the relationship between insulin resistance (as quantified by HOMA-IR) and the free androgen index (FAI) [31]. Notably, a positive correlation was observed between circulating dynorphin levels and the LH/FSH ratio, whereas no such association was identified between kisspeptin and the LH/FSH ratio [32].

There are contradictory results from the information on plasma prolactin levels in PCOS-afflicted women [33]. Occurrence of hyperprolactinemia (HPRL) linked to PCOS has not been consistently established by recent research [34]. A considerable percentage of PCOS individuals having higher prolactin levels remained initiate to have extra coexisting features accountable for the high level of prolactin after utilizing a broad investigative method, which includes looking through possible sources of raised prolactin levels, comprising of prolactin chromatography and pituitary gland imaging, and eliminating other features, comprising reduce thyroid level, gestation, and the usage of medications that may uplift prolactin [35]. In order to establish the correlation, a retrospective study was conducted in which a substantial elevation in the level of serum prolactin was detected [36].

PCOS shares clinical symptoms with certain thyroid conditions. PCOS and hyperthyroidism seldom co-occur; the thyroid conditions most commonly linked to PCOS are subclinical hypothyroidism (SCH) and Autoimmune Thyroid Diseases (AITDs) [37]. This correlation implies that the thyroid may influence several systems, such as metabolism and immunity, and hence impact the clinical signs of PCOS [38]. Thyroid function plays a critical role in modulating insulin sensitivity; notably, hypothyroidism is associated with more severe insulin resistance compared to euthyroid states [39]. Given that insulin resistance is a well-established etiological factor in PCOS [40], one study reported a significantly higher prevalence of thyroid dysfunction among individuals with PCOS, highlighting a potential pathophysiological link between these two endocrine disorders [41].

The main issue in the clinical assessment of PCOS is the genetically determined excess androgen release from the ovary [42]. Early androgen productions are typically regarded as premature in PCOS and is believed to play a role in insulin resistance development [43]. Insulin resistance in visceral adipocytes is caused by disruptions in lipid metabolism [44]. However, the progression of insulin resistance is directly as well as specifically influenced by excessive testosterone exposure [45]. Increased testosterone secretion is linked to islets of Langerhans dysfunction, which impairs pancreatic metabolic processes and results in hyperinsulinemia [46]. It was identified as a primary cause of type-2 diabetes in preclinical research with females suffering from PCOS [47].

### 4.2 Metabolic Markers in PCOS

Hypothalamus-hypophysis-ovary axis features seem disturbed *via* insulin-compromised of biochemical signaling. The altered composition and functionality of muscle tissue and increasing amounts of free fatty acids, androgens can then cause insulin resistance (IR) again, extending the cycle of IR, hyperinsulinemia, and hyperandrogenemia [48]. Women with polycystic ovary syndrome (PCOS) who have low adiposity exhibit unique hormonal and biochemical profiles [49]. Despite lower body fat, both lean and obese women with PCOS are susceptible to chronic cardiometabolic comorbidities, including type 2 diabetes mellitus, dyslipidemia, metabolic syndrome, and cardiovascular disease-conditions that are often driven by underlying chronic inflammation [50]. Given these serious health implications, it is critical to fully elucidate the complex pathophysiological mechanisms underlying PCOS in order to develop targeted therapeutic strategies and improve the quality of life for affected individuals [51]. Interestingly, no clear distinction was found between insulin-resistant and non-insulin-resistant PCOS patients, although modest correlations with insulin resistance markers were observed [52]. Perimenopausal women who had previously had a wedge resection for polycystic ovarian syndrome, 32% had type 2 diabetes [53]. Reproductive problems may occur during puberty, depending on when diabetes was initially diagnosed [54]. Due to central hypogonadism, women with type 1 diabetes have historically had both infertility and amenorrhea [2]. Better metabolic control and more effective insulin administration have decreased the problems, but not completely disappeared [55]. Family physicians face difficulties in treating type 2 diabetes, a prevalent and often disregarded condition [56]. All individuals diagnosed with PCOS between the ages of 16 and 40 were included in the cross-sectional investigation. Up to 37.4% of people with PCOS have decreased glucose tolerance. The development of decreased glucose tolerance in females having PCOS is significantly predicted by growing ovarian volume, elevated serum testosterone levels, and rising age and body mass index [57].

### 4.3 Role of Stress in PCOS

Emerging evidence suggests that chronic psychological stress may play a contributory role in the development and progression of PCOS. Stress activates the hypothalamic pituitary adrenal (HPA) axis [58], leading to increased cortisol production [59], which can disrupt gonadotropin-releasing hormone (GnRH) [58] and promote insulin resistance [60]. These alterations may further exacerbate hyperandrogenism [61] and menstrual irregularities [62] in individuals with PCOS. Although still under investigation, addressing stress through behavioral or psychological interventions may represent a complementary strategy for managing PCOS symptoms.

## 5 DIAGNOSTIC CRITERIA

The diagnosis of PCOS is based on the dynamic presence of three key features: oligo or anovulation, hyperandrogenism (clinical or biochemical), and polycystic ovarian morphology as assessed by transvaginal ultrasonography [63]. The Rotterdam criteria call for the occurrence of two of the three investigative standards in order to diagnose mature females, which were approved through the Global Evidence-based Recommendation [64]. In individuals who exhibit amenorrhea and other unusual characteristics, additional assessment is advised [65]. Hypogonadotropic hypogonadism or Cushing syndrome should be excluded [66]. Plasma androgen levels that remain two times the higher confines of usual are aimed at native scientific assessment, regularly indicating presence of severe androgenic profiles [67]. The diagnostic features of polycystic ovary syndrome (PCOS) vary across ethnic groups and throughout different life stages, thereby complicating its classification and the understanding of its natural history [68]. Clinical or biochemical methods can be used to evaluate androgenic status [69]. Skin conditions, including hirsutism, pimples, or baldness, are clinical indicators of androgenic excess [70]. Clinical androgenic expression varies greatly among ethnic groups, and self-treatment of hirsutism frequently restricts examination [71].

Table **1** delineates the evolution of diagnostic criteria for PCOS across three major standards: the Androgen Excess Society (2006), Rotterdam Criteria (2003), and National Institutes of Health (1990). Hyperandrogenism, defined clinically (hirsutism, acne, androgenic alopecia) or biochemically, is a central component in all criteria but is mandatory only in the NIH and Androgen Excess Society guidelines. Ovulatory dysfunction is essential in the NIH criteria but optional in the Rotterdam model, which allows diagnosis with two of three features. Polycystic ovarian morphology is integral to Rotterdam but not mandatory elsewhere. All criteria necessitate exclusion of differential diagnoses such as thyroid dysfunction and congenital adrenal hyperplasia. The Androgen Excess Society mandates hyperandrogenism alongside either ovulatory dysfunction or ultrasonographic evidence of polycystic ovaries, while Rotterdam adopts a broader approach. Overall, the criteria have shifted from stringent definitions to a more inclusive framework, enhancing diagnostic sensitivity for diverse PCOS phenotypes.

## 6 DEMOGRAPHIC PARAMETERS IN PCOS

### 6.1 Ethnicity and Genetic Factors: Variations in PCOS Prevalence & Phenotype

The possibility that the phenotypes of PCOS reflect a mismatch between ancient metabolic and reproductive survival systems that are genetically encoded and contemporary lifestyle choices has gained more attention [87]. When exposed to particular postnatal settings, gene variations that incline the progenies towards the progression of PCOS may be activated by in-utero developmental programming of metabolic as well as endocrine pathways [88]. Reported pathophysiological changes and clinical characteristics seen in women with PCOS may be the consequence of epigenetically programmed pathways that are modulated by postnatal exposure to lifestyle variables such as poor diet and endocrine-disrupting substances (Table **2**) [89].

Thus far, four distinct phenotypes of PCOS have been found, and they are as follows: Types A, B, OD, and D, including PCO, chronic anovulation HA, and hyperandrogenism (H), polycystic ovaries (PCOM), and hyperandrogenism (H), as well as PCO plus chronic anovulation (CA), respectively [90]. Since the majority of PCOS women are obese with severe or mild metabolic deformities and frequently struggle with dyslipidemia, hyperinsulinemia, insulin resistance, and other metabolic abnormalities, the category of polycystic ovary syndrome is linked to metabolism as well as cardiovascular health [91]. The main topic of debate is the fabrication of a link between aging and PCOS characteristics [92]. Compared to phenotypic B, patients having phenotype A were found to have higher levels of insulin resistance, also to have been exposed to more androgens [93]. Even in the absence of excessive androgen exposure, phenotype D is typically defined by insulin resistance in obese individuals [94]. The situation is different for type C, where there is a substantial risk of cardiovascular disease, which may be brought on by PCOS's lack of insulin resistance [95].

### 6.2 Socioeconomic Status: Access to Healthcare and Diagnosis Rates

One frequent endocrinopathy that is very common among Indian women is polycystic ovarian syndrome, or PCOS [100]. Ethnic and cultural variations in analysis as well as management of polycystic ovary syndrome in women are not sufficiently taken into account by clinical practice recommendations [101]. An international online survey was conducted among reproductive-aged ethnic Indian women diagnosed with polycystic ovary syndrome (PCOS). The mean age of respondents (n = 4,409) was 26.8 years (SD = 5.5). A family history of PCOS and type 2 diabetes mellitus was reported by 18% and 43% of participants, respectively. Notably, 64% had been diagnosed with at least one comorbidity, with anxiety and depression being the most common. The three primary concerns reported were hirsutism (excessive facial hair), ovarian cysts, and menstrual irregularities. On average, women experienced initial PCOS symptoms at 19.0 years (SD = 5.0) and received a formal diagnosis by 20.8 years (SD = 4.8). Dissatisfaction with the quality and clarity of information provided about PCOS and its treatment options was significantly associated with a diagnostic delay of approximately seven months and a one-year delay in seeking medical care (*p* < 0.01). Most respondents also reported poor quality of life, particularly related to weight and emotional well-being. These findings suggest that PCOS symptoms manifest early among ethnic Indian women, yet timely access to specialized care is often delayed [102].

## 7 LIFESTYLE FACTORS

### 7.1 Obesity

**Fig. (3):**
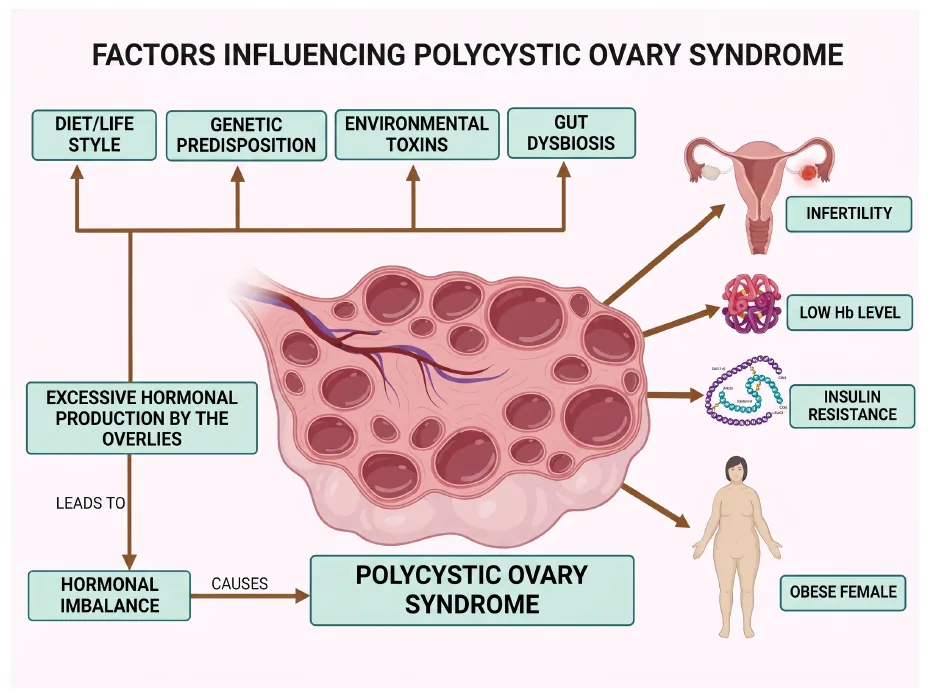
The figure represents the factors impacting Polycystic Ovary Syndrome (PCOS), which includes contributing factors (diet/lifestyle, genetic liability, environmental toxins, and gut imbalance), core state (presented by hormonal interruption and ovarian impairment), and related health outcomes (infertility, low haemoglobin, insulin resistance, and obesity).

Obesity is the most prevalent reproductive condition affecting women who are known to have PCOS [103]. Since many females having PCOS are known to be obese, one of the major contributing factors in the advancement of this condition [104]. In an attempt to establish if PCOS causes obesity or whether obesity causes biochemical abnormalities that consequence in PCOS, some research examines the link between PCOS and obesity [105]. Numerous studies on the subject were reviewed and compared to conduct the analysis. Both obese and non-obese PCOS participants had their body fat distribution, insulin resistance, and hyperandrogenism analyzed [106]. But none of the studies were able to definitively establish whether obesity was caused by PCOS or *vice versa* (Fig. **3**) [107].

### 7.2 Diet

 Research has evaluated how well women with PCOS regulate their caloric intake and expenditure, but the findings have been mixed [108]. It is challenging to compare research because of the limited sample size, the variability of the study participants, and the various BMI classifications [109]. Some research showed that females suffering from PCOS consumed more energy plus superior-quality foods, while other studies found no changes in everyday energy consumption among females with as well as without PCOS. Furthermore, not much research has been done on the eating habits of women with PCOS, such as restricted eating plus sensitive eating. Specifically, “obesogenic society”, emotional as well as outdoor eating behaviors may impact food consumption, increasing the likelihood of weight gain [110].

### 7.3 Urban *vs.* Rural

Living in nuclear households in metropolitan regions was associated with a higher occurrence of PCOS than in rural areas [111]. Among the urban participants, there was a higher prevalence of oligomenorrhea along with recent weight increase and obesity [112]. Higher levels of serum insulin were linked to elevated androgen action, as shown by amplified hirsutism, in urban regions than opposed to rural ones [113]. Associated with the rural crowd, the urban crowd had a greater prevalence of PCOS symptoms. Lack of exercise, an obesogenic diet, and obesity, environmental factors of PCOS, were more common in urban groups than in rural ones [114].

## 8 INTERRELATIONSHIP BETWEEN BIOCHEMICAL AND DEMOGRAPHIC PARAMETERS

### 8.1 Correlation between Insulin Resistance and Obesity

Overconsumption of energy causes cell overload, which sets off defense mechanisms that lower insulin sensitivity and shield cells from more energy buildup [115]. Furthermore, local inflammation brought on by hypertrophied adipocytes and macrophage infiltration leads to systemic inflammation, which in turn triggers IR [116]. Clinical manifestations range from metabolic diseases like Metabolically Associated fatty liver disease or diabetes mellitus to skin lesions like acanthosis nigricans [117]. The sensitivities and specificities of the many IR laboratory indicators differ. Since a decrease may cause the inflammatory state to revert in fat tissue, plus also lessens the degree of resistance to insulin, dietary modifications and consistent physical exercise are essential for managing insulin resistance [118]. PCOS in relation to insulin resistance is also mentioned as a disorder with a genetic component to its development and regarded as a hereditary condition [119], which has ethnic and genetic variations on biochemical markers [120].

### 8.2 Impact of Ethnic and Genetic Variations on Biochemical Markers

The study found that PCOS affects 6% to 9% of people in the USA, Spain, Brazil, Mexico, Iran, Africa, and Asia. Using the National Institute of Health criteria, it was suggested that Chinese females had the lowest prevalence of PCOS, whereas Qatari females had a higher prevalence than the Caucasian population. According to this study, the prevalence of PCOS is influenced by racial or ethnic factors to a moderate to high degree [18]. The possibility that the phenotypes of PCOS reflect a mismatch between ancient metabolic and reproductive survival systems that are genetically encoded and contemporary lifestyle choices has gained more attention [121].

### 8.3 Lifestyle Factors on Hormonal and Metabolic Profiles

In PCOS, abnormal gut hormone regulation and energy expenditure may affect physical capacity for lifestyle adjustments [122]. This could make it more difficult to control weight. Additional obstacles include the increased incidence of eating disorders, disordered eating, exhaustion, and sleep difficulties [123]. Due to psychological symptoms and a lack of critical health literacy, psychological capabilities are diminished [124]. Women with PCOS are experiencing pathological symptoms due to hormonal abnormalities. It has been shown that PCOS causes a variety of hormone changes [125]. PCOS women have aberrant levels of several hormones, including the luteinizing hormone/follicle-stimulating hormone (LH/FSH) ratio, androgens, oestrogens, ghrelin, LEAP-2, gonadotropin-releasing hormone (GnRH), insulin, and growth hormones (GH). These hormones are linked to metabolic conditions like insulin resistance and diabetes, obesity and overweight, infertility, and irregular menstruation in PCOS patients. PCOS females are more likely to have diabetes and obesity due to the obsessive alterations of the hormones, which include elevated insulin, decreased growth hormones, increased ghrelin, as well as leptin resistance. PCOS is associated with infertility and is characterized by decreased GH, elevated LEAP-2 levels, high basal luteinizing hormone, elevated luteinizing hormone/follicle-stimulating ratio, increased androgens, and decreased estrogen [19]. Lifestyle factors cause insulin resistance and obesity, which leads to PCOS, causing genetic variations and runs in family histories [42].

## 9 CLINICAL IMPLICATIONS AND MANAGEMENT

### 9.1 Relevance of Biochemical and Demographic Evaluation for Personalized Treatment

Treatment alternatives for PCOS metabolic profile comprise diet, exercise, weight loss, bariatric surgery, insulin sensitizers, liraglutide, and statins [126]. Weight loss, gonadotropins, letrozole, clomiphene citrate, oral contraceptives, and laparoscopic ovarian drilling are all part of the treatment strategy for PCOS's reproductive phenotype. PCOS's hyperandrogenic phenotype is treated with eflornithine hydrochloride, GnRH-agonists, oral contraceptives, antiandrogens, and cosmetic treatments. Alternative treatments for PCOS have surfaced, such as acupuncture, dietary changes, and the use of herbs and supplements [127].

### 9.2 The Role of Lifestyle Interventions and Pharmacological Treatments

According to global evidence-based guidelines for PCOS, managing weight and lifestyle factors (diet, exercise, and behavior) is the first line of treatment [122]. The possible advantages of combining various traditional, complementary, and integrative medicine (TCIM) techniques with psychiatric and sleep therapies are a topic of ongoing interest and research [128], for the best management of PCOS, even though this advice adheres to population-level dietary and physical activity guidelines [129]. Insufficient data to suggest a particular diet for PCOS, with strategies such as altering the amount or quality of fat, protein, or carbohydrates generally having comparable impacts on PCOS symptoms [128]. In terms of physical activity, there is encouraging evidence that providing intense aerobic exercise can improve insulin resistance, body composition, and cardiorespiratory fitness [130]. Given that women have lower rates of clinical and subclinical sleep disturbance and poorer emotional well-being, psychological and sleep therapies are especially crucial since they may impede their capacity to adopt healthy lifestyle choices. Although improving sleep and mental health may help manage PCOS symptoms, there is a dearth of information on the effectiveness of clinical treatments. Women with PCOS are increasingly using TCIM strategies, including the use of supplements and herbal medicines. However, integration into standard clinical practice is not yet supported by enough data. The most encouraging results from studies looking into inositol supplementation have shown decreased hyperandrogenism and enhanced metabolic profiles [131].

## 10 METHODOLOGICAL APPROACHES IN EVALUATING PCOS

### 10.1 Techniques for Measuring Biochemical Markers

Depending on the clinical presentation, blood tests are used to measure biochemical markers in PCOS [132]. These tests typically measure hormone levels, especially androgens like testosterone (total and free), and frequently include measurements of sex hormone-binding globulin to determine the Free Androgen Index, along with other hormones like LH, FSH, and DHEAS [133]. Additional tests measure insulin and fasting glucose levels to assess insulin resistance, which is frequently linked to PCOS [52]. Ideally, blood should be drawn on the second to third day of the menstrual cycle for accurate hormone level evaluation [134]. Using high-quality assays like LC-MS/MS is crucial for accurate androgen measurement [135].

## 11 LIMITATIONS AND GAPS IN EXISTING RESEARCH

In precisely defined study groups, metabolic syndrome and obviously overactive immune systems must be given more research focus since they represent distinctly separate hereditary characteristics [136]. It will be challenging to make additional advancements in our understanding of PCOS if these two disorders cannot be accurately distinguished from one another [137]. The main drawback of this study is the presumption that patient self-selection is the individual description for extremely unique spreading of PCOS patients in our institution, according to the Rotterdam criteria [138]. One must also acknowledge that the reason for this self-selection at our center is that patients with phenotypes A, B, and C did not have treatment failures elsewhere if [139], we concluded that there is no alternative reason why our center has seen a large number of females with phenotypic D, PCOS in recent years while seeing very few patients with phenotype A, B, and C [140]. Naturally, more treatment failures indicate that phenotypic D patients are more resistant to treatment, which is addressed by testosterone supplementation, as this case-control study showed [141]. Thus, this work provides a potentially novel view of PCOS, proposes that 1. Hyperandrogenic hypoovulatory PCOS is an underdiagnosed health condition, particularly in females over 35, as well as provides a straightforward therapeutic option for these individuals by using androgen supplements [12].

## 12 EMERGING TRENDS AND FUTURE DIRECTIONS

### 12.1 Advances in Biochemical Resting (*e.g., Biomarkers for Early Detection*)

Both reproductive and non-reproductive issues, such as obesity, diabetes, insulin resistance, dyslipidemia, and elevated blood pressure, are linked to PCOS [142]. Biomarkers that target the reproductive and non-reproductive components of this complicated disease are desperately needed [143]. This review concentrates on biomarkers or possible biomarkers associated with PCOS's reproductive and non-reproductive features. These consist of lipids, insulin, and the insulin-like growth factor 1 system, clinical and anthropometric biomarkers, steroids, gonadotropins, and anti-Müllerian hormone, biomarkers for kidney injury and inflammation, oxidative stress, and noncoding RNAs [144].

### 12.2 Precision Medicine Approaches for Demographic-Specific Interventions

Hormonal dysregulations should be the primary objective of PCOS treatment [145]. Furthermore, patients from different ethnic and geographical backgrounds may respond differently to PCOS therapy, which emphasizes the need for customized treatment regimens for better PCOS control [146]. Changing one's lifestyle is the primary line of treatment for PCOS [147], which includes reducing daily caloric intake, engaging in regular exercise, taking medication, and, in cases of morbid obesity, having weight loss surgery or occasionally bariatric surgery [148]. Ovulation initiation with anti-estrogen therapy using clomiphene citrate is one pharmacological therapeutic option [149]. This is the conventional first-line treatment for anovulatory PCOS, which increases gonadotropin release and stimulates the growth of ovarian follicles [150]. However, for better results, Sensitizer for insulin Metformin, either alone or in combination with letrozole or clomiphene citrate, can also be used to stimulate ovulation [151]. The use of clomiphene citrate may soon be replaced by letrozole, which was suggested as a first-line pharmaceutical therapy for the stimulation of ovulation [152]. However, Letrozole and clomiphene citrate together may work better than letrozole by itself [153]. Furthermore, in patients having unsuccessful ovulation as well as are not responsive to oral medications, gonadotropins constitute the secondary treatment for ovulation induction [154]. In treating clomiphene-citrate-resistant PCOS [155], another management technique called laparoscopic ovarian drilling is just as successful as gonadotropins [156]. Those who need laparoscopic pelvic evaluation, have a high LH index, or are unable to consistently monitor gonadotropin therapy are more likely to benefit from it [157]. It entails laparoscopically puncturing the membranes around the ovary to induce ovulation [158]. However, patients may be directed to assisted reproductive technology (ART) if first-line or second-line medication for ovulation induction proves ineffective [159]. Nonetheless, a number of possible genetic factors have now been discovered through genome-based research to enhance PCOS screening plus treatment facilities that exhibit greater promise and effectiveness [160].

### 12.3 Integration of Artificial Intelligence (AI) and Big Data for Better Evaluation

Recently, the healthcare sector has used AI technology as a common approach for managing and diagnosing complicated disorders like PCOS [161]. AI swiftly and correctly diagnoses PCOS by analyzing ultrasound images, anthropometric data, and biochemical test results using machine learning algorithms [162]. Lab results, medical records, and patient histories are just a few of the data sources that AI may assist in integrating to create a thorough and understandable picture of an individual's health [163]. The doctor can use this information to make more effective and knowledgeable diagnostic choices [164]. With an emphasis on AI-based diagnostic tools, this review paper offers a thorough examination of the developing role of AI in several facets of PCOS care [165].

### 12.4 Precision Medicine & Emerging Therapies

In contrast, gene therapy attempts to address the underlying genetic reasons of PCOS, which may provide a more fundamental and focused remedy for hormone imbalance [166].

A number of genes, including CYP11a, CYP21, CYP17, and CYP19, have been linked to the making plus control of steroid hormones [167]. In steroidogenesis, for example, an enzyme required for the conversion of cholesterol to progesterone is encoded by CYP11a [168]. Although CYP11a polymorphisms have been connected to PCOS in certain investigations, these links have not been reliably confirmed in other studies [169]. Similarly, there is conflicting evidence about the direct relationships between CYP21, which converts 17-hydroxyprogesterone to 11-deoxycortisol, and PCOS-like symptoms, particularly in cases of hyperandrogenism [170]. Many studies are interested in the dysbiosis of the GMB in females suffering from PCOS [171]. Porphyromonas species, *B. coprophilus*, and *F. prausnitzii* are more common in the stomachs of women with PCOS.

Studies conducted recently have shown that miRNAs can be found in a variety of bodily fluids, including the follicular fluid of PCOS-affected women [172]. As a result, it might function as a novel therapeutic target and a possible biomarker for PCOS diagnosis and treatment [173].

Hyperandrogenism is linked to gut microbial dysbiosis, suggesting that androgens can alter the gut microbial population in women [174], according to research done on PCOS-afflicted humans and rodent models [175]. Acupuncture, herbal remedies, and vagus nerve stimulation have been utilised recently to treat PCOS and control hormone levels [176].

## CONCLUSION

The diagnosis of PCOS remains controversial due to inconsistent androgen thresholds, subjective assessment of clinical features such as hirsutism, variation in ultrasound interpretation of polycystic ovarian morphology, and over-reliance on menstrual irregularity, all of which contribute to diagnostic inconsistencies across populations and healthcare systems. The cited literature reveals limitations, including non-uniform diagnostic criteria, heterogeneous study designs, small sample sizes, and insufficient long-term data. These gaps hinder a comprehensive understanding of PCOS and delay the development of effective, personalised treatment strategies. Future research should prioritize identifying at-risk individuals using genomic and proteomic tools, which also examine the genetic and environmental factors that contribute to PCOS onset and variability across diverse groups.

## Figures and Tables

**Fig. (2):**
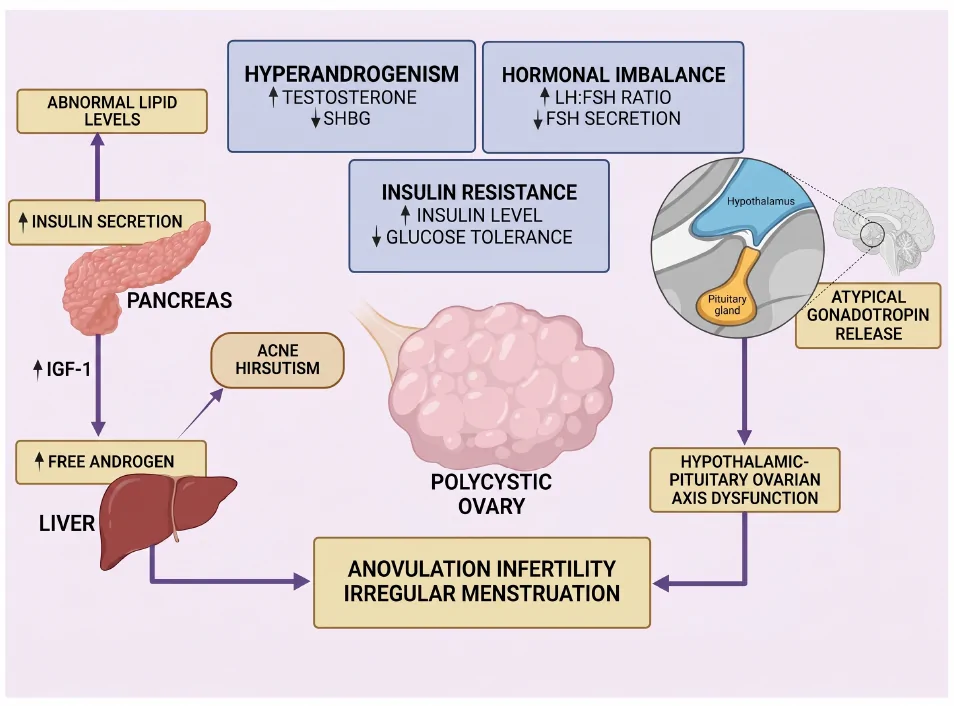
The above figure depicts the complicated interplay providing to Polycystic Ovary Syndrome (PCOS) detailed as primary role (resulting in the symptoms such as anovulation, infertility and irregular menstruation.), hormonal instability (hyperandrogenism which is increased level of testosterone and reduction in sex hormone-binding globulin yielding to symptoms like acne and hirsutism, modification in the luteinizing hormone to follicle-stimulating hormone FSH ratio and lowered FSH secretion.), insulin resistance (raised levels of insulin and hindered glucose tolerance.), free androgens and IGF-1 (amplified levels of free androgen and IGF-1 extended from the liver which may worsen the disease condition, polycystic ovary (in case of PCOS ovaries may increase in size leads to multiple cyst resulting in anovulation, infertility, irregular menstruation), hypothalamic-pituitary ovarian axis dysfunction (the disturbance in the transmission between the hypothalamus, pituitary gland, ovaries leads to lack of ovulation and may also cause infertility.

**Table 1:** Development of PCOS diagnostic standards.

**Criteria**	**Androgen Excess Society (2006)**	**Rotterdam Criteria (2003)**	**National Institutes of Health (1990)**
**Hyperandrogenism**	Diagnosis requires clinical or biochemical hyperandrogenism [72].	One of the three requirements is clinical or biochemical hyperandrogenism, but it is not necessary [73].	Clinical (hirsutism, acne, androgenic alopecia) or biochemical evidence of hyperandrogenism required [74].
**Ovulatory** **Dysfunction**	Required, along with hyperandrogenism [75].	One of the three criteria is chronic anovulation or irregular cycles, but it is not necessary [76].	Unusual cycles or persistent anovulation (such as amenorrhea or oligomenorrhea) [77].
**Polycystic Ovarian Morphology**	Supports diagnosis but is not required [78].	Existence of ≥ Twelve (2–9 mm) follicles in one or both ovaries with a volume of more than 10 mL is one of three criteria [79].	Not required [80]
**Exclusion of Other Disorders**	Required to rule out other conditions [81].	Required to rule out other causes of hyperandrogenism or anovulation [82].	Required (*e.g*., thyroid dysfunction, hyperprolactinemia, congenital adrenal hyperplasia) [83]
**Diagnostic Standards**	Diagnosis requires hyperandrogenism & either ultrasonography findings of polycystic ovaries or ovulatory dysfunction [84].	Analysis requires 2 of 3 criteria: An elevated level of androgen & ovulatory abnormality or polycystic ovaries on ultrasound [85].	Both hyperandrogenism & ovulatory dysfunction must be present [86].

**Table 2:** Phenotype of polycystic ovary syndrome.

**Phenotype**	**A**	**B**	**C**	**D**
**Hyperandrogenism (HA)**	Existing	Existing	Existing	Non-Existing
**Ovulatory Dysfunction (OD)**	Existing	Existing	Non-Existing	Existing
**Polycystic Ovarian Morphology (PCOM)**	Existing	Non-Existing	Existing	Existing
**Characteristics**	Most severe; consists of all three features [96].	Severe; consists of HA & OD but normal ovaries [97].	Milder; consists of HA & PCOM but regular cycles [98].	Milder; consists of OD & PCOM without hyperandrogenism [99].

## LIST OF ABBREVIATIONS

PCOS: Polycystic Ovary Syndrome
AI: Artificial Intelligence
IR: Insulin Resistance
CYP11A: Cytochrome P450 Family 11 Subfamily A
CYP21: Cytochrome P450 Family 21
CYP17: Cytochrome P450 Family 17
CYP19: Cytochrome P450 Family 19
P450scc: Cytochrome P450 Side-Chain Cleavage Enzyme
BMI: Body Mass Index
FSH: Follicle-Stimulating Hormone
LH: Luteinizing Hormone
GH: Growth Hormones
GnRH: Gonadotropin-Releasing Hormone
LEAP-2: Liver-Expressed Antimicrobial Peptide 2
SHBG: Sex Hormone-Binding Globulin
AMH: Anti-Müllerian Hormone
DHEAS: Dehydroepiandrosterone Sulfate
FT: Free Testosterone
FAI: Free Androgen Index
IGF-1: Insulin-Like Growth Factor 1
NIH: National Institutes of Health
HA: Hyperandrogenism
OD: Ovulatory Dysfunction
PCOM: Polycystic Ovarian Morphology
AITDs: Autoimmune Thyroid Diseases
SCH: Subclinical Hypothyroidism
PCO: Polycystic Ovaries
CA: Chronic Anovulation
SD: Standard Deviation
HPRL: Hyperprolactinemia
T2DM: Type 2 Diabetes Mellitus
IVF: *In Vitro* Fertilization
ART: Assisted Reproductive Technology
HOMA-IR: Homeostatic Model Assessment for Insulin Resistance
SDI: Socio-Demographic Index
TCIM: Traditional Complementary Integrative Medicine
LC: Liquid Chromatography
MS: Mass Spectrometry
HH-PCOS: Hyperandrogenic Hypo-Ovulatory Polycystic Ovary Syndrome
GMB: Gut Microbiota

## STANDARDS OF REPORTING

PRISMA guidelines and methodologies were followed.

## SUPPLEMENTARY MATERIAL

PRIMSA checklist is available as supplementary material along with the published article.
